# Utility of Molecular Identification and Quantitation of *Bartonella* Species with Species-Specific Real-Time PCR for Monitoring Treatment Response: A Case Series

**DOI:** 10.2174/1874285801812010148

**Published:** 2018-05-31

**Authors:** Maria Mazzitelli, Angelo G. Lamberti, Angela Quirino, Nadia Marascio, Giorgio S. Barreca, Chiara Costa, Vincenzo Pisani, Alessio Strazzulla, Giuseppe Greco, Maria C. Liberto, Alfredo Focà, Carlo Torti

**Affiliations:** 1 Infectious and Tropical Diseases Unit, Department of Medical and Surgical Sciences, “*Magna Graecia*” University of Catanzaro, Viale Europa, 88100, Catanzaro, Italy; 2 Institute of Microbiology, Department of Health Sciences, “*Magna Græcia*” University, Viale Europa, 88100, Catanzaro, Italy

**Keywords:** Molecular identification, Quantitation, *Bartonella* species, Intracellular bacteria, Trench fever, Real time PCR

## Abstract

**Background:**

Bartonella species are intracellular bacteria capable of producing several diseases in humans. The three most common and wellknown diseases are cat scratch disease (CSD), caused by B. henselae, trench fever, caused by *B. quintana* and Carrion’s Disease, caused by *B. bacilliformis*. Signs and symptoms are very different and aspecific: Fatigue, fever, headache, lymphadenopathy, malaise, loss of weight. No data exist to support guidelines’ recommendations to decide which drugs should be optimally used and how long they should be administered. Therefore, a marker of treatment response is needed to guide treatment strategies.

**Methods:**

We report herein three cases in which a species specific Reverse-Transcriptase Polymerase-Chain-Reaction (RT PCR) developed in-house was performed and compared to serology in order to make diagnosis and to evaluate treatment response.

**Results:**

Our species-specific RT PCR seemed to play a fundamental role both in diagnosis and treatment. Moreover, a discrepancy with the serology results was found.

**Conclusion:**

Further studies are necessary to validate these results and elucidate what is the best treatment for this pleomorphic disease. However, in absence of clear guidelines, RT PCR may be useful to orientate kind of treatment ad its duration.

## 1 BACKGROUND

*Bartonella* species are intracellular bacteria capable of producing several diseases in the human host. The three most common and well-known diseases are Cat Scratch Disease (CSD), caused by *Bartonella henselae* (*B. henselae*), trench fever, caused by *Bartonella quintana* (*B. quintana*) and Carrion’s Disease, caused by *Bartonella bacilliformis*. (*B. bacilliformis*) Members of the *Bartonella* genus are also capable of producing chronic bacteraemia in human hosts, bacillary angiomatosis, peliosis hepatitis, neurological disorders, endocarditis, myocarditis, retinitis and chronic lymphadenopathies. Signs and symptoms are very diverse and aspecific: fatigue, fever, headache, lymphadenopathy, malaise, and loss of weight [1]. No data exist to support guidelines’ recommendations to decide which drugs should be optimally used and how long they should be administered [2]. Therefore, a marker of treatment response is needed to guide treatment strategies.

We report herein three cases in which a homemade Real-Time Polymerase Chain Reaction (RT PCR) seemed to play a fundamental role both for diagnosis and treatment monitoring.

## 2 MATERIALS AND METHODS 

We collected the serological and molecular biology data of three patients with bartonellosis followed at the outpatient clinic of Infectious and Tropical Diseases Unit of “*Mater Domini*” teaching hospital in Catanzaro (Italy). We compared, in particular, the concordance of serological test results (IgG and IgM for *B. henselae*) with the result of real-time PCR, and clinical outcome after therapy.

*B. henselae* IgG and IgM were tested by indirect fluorescent antibody analysis (Euroimmun, Medizinische Labordiagnostika AG, Germany) using a IgG titre ≥1:64 and IgM titre ≥1:20 as cut-off, according to the manufacturer’s instruction.

A homemade real-time PCR with SYBR Green 1 dye was performed as previously described [3, 4]. Briefly, DNA extraction from whole blood was carried out using the QIAamp DNA Blood Mini Kit (QIAGEN^®^ Group) according to the manufacturer’s instruction. Two μl of extracted DNA (20 ng) were used for the amplification by real-time PCR with SYBR Green 1 dye using a pair of primers (Forward 5’-TTGCGACAAAACAGCTTCAC-3' and Reverse 5’-GATGGAGCATCTGCACTCAA-3’), which amplify a 510 bp region of the *bqtR* gene. *B. henselae* melting temperature (Tm) was 87.39±0.20° C. We amplified a 510 bp fragment of the *bqtR* gene because it was able to distinguish *B. quintana* and *B. henselae* strains by melting temperature. The melting temperatures were significantly (P<0.05) different, therefore allowing us to discriminate the two species (3,4). Amplification and melting curve of *B. henselae* can be seen in Fig. (**1**).

Diagnostic workup was completed with RT PCR as soon as we received positive results of IgG for *Bartonella*. Therapy was started as soon as patients were contacted and came back to consultation after receiving positive RT PCR.

## 3 CASE REPORTS

### 3.1 Case Report #1 (Table **1**)

A 42 years-old Caucasian male (weight: 84 kg, height: 178 cm), came to our observation in July 2013 complaining about asthenia associated with a right latero-cervical lymphadenomegaly, started two years before. The patient, along with these symptoms, has never reported fever. He was resident in a rural area, living in contact with several animals (in particular birds and cats) and worked as a volunteer in a human hospital. The patient reported allergy to diclofenac, *cipressaceae* and *parietaria* plants. He underwent two interventions for inguinal hernia, the first at the age of 11 years and the second at the age of 34. In 2004, he suffered from kidney stones. In 2004 he had toxoplasmosis. In July and in August 2014 he was referred twice to the Unit of Neurology because of a previous diagnosis of epilepsy treated with levetiracetam and topiramate from 2002 to 2007. Upon admission to the neurology ward, serological tests for several agents were performed (*T. gondii*, *Cytomegalovirus*, Epstein-Barr virus, *Brucella*, HBV, HCV, HIV, *T. pallidum*, *Salmonella* spp.), and all were negative. Full blood cell count was normal. Lymph node ultrasound (US) documented the presence of numerous lymph nodes at submandibular, lateral cervical, axillary and groin regions with maximum diameter of about 2.5 cm. So, we decided to perform both serology and RT PCR for *Bartonella spp*., for the suspicion of CSD. The result of a positive RT PCR for *B. henselae* led us to star therapy with doxycycline 100 mg, twice daily, *per os* for ten days. Then the patient discontinued treatment for epigastralgia. After 9 days, he restarted doxycycline 100 mg, twice daily, *per os*. We suggested him, in order to avoid further epigastralgia, to take a gastroprotective therapy, so he was able to continue an appropriate treatment for two weeks. This therapy was stopped after RT PCR for *B. henselae* DNA resulted to be undetectable. After antibiotic therapy, there was a significant reduction of lymphadenopathy and fatigue and asthenia recovered. At the last outpatient consultation, in June 2014, just a small left later cervical node was evident, followed by complete regression in January 2015.

### 3.2 Case Report #2 (Table **2**)

A 69 years-old female, with hypercholesterolemia on treatment, came to our observation in August 2013 for right axillary lymphadenopathy just started, associated with recurrent fever, chilling and malaise. These symptoms appeared 4 months before. She lived, like patient #1, in a rural area and was in contact with cats and rabbits in her countryside village. Full blood cell count was normal. Also in this case serologies for several infections were performed (*T. gondii*, *Cytomegalovirus*, Epstein-Barr virus, *Brucella*, HBV, HCV, HIV, *T. pallidum*, *Salmonella* spp.), and all were negative. Furthermore, both serology and RT PCR for *Bartonella* spp. were performed. In particular, IgG were positive (with 1:320 titre), IgM were negative and RT PCR for *B. henselae* DNA was positive. Chest X-ray did not show any abnormalities. Lymph node US revealed an enlarged lymph node in the right axilla with thickened cortical region with a size of 1.5 x 1 cm. So, in addition to the serological tests, the patient underwent incomplete removal of the axillary lymph node that appeared partially necrotic. Histopathological examination of the lymph node showed: “Granulomatous lymphadenitis in epithelioid cells with florid hyperplasia and areas of necrosis”. After biopsy, drainage of purulent material persisted. The patient started a treatment with intravenous (IV) gentamicin 80 milligrams three times per day for a week in addition to IV ceftriaxone 2 g *per* day for a week, then continued treatment with doxycyclin 100 mg *per os* twice daily for 6 weeks. At the last check, the patient reported well-being, no fever and just a residual pain at the site of the biopsy.

### 3.3 Case Report #3 (Table **3**)

A 35 years-old Caucasian male, smoker and working as a bricklayer, was living in contact with different animals (cats, dogs and parrots). He came to our observation for asthenia, lymphadenopathy in left axillary region, arthralgia and low-grade fever in the evening, lasting for two months. He referred many cat scratches from his cats and many insect bites four months before symptom begun. Serological tests for several agents appeared to be negative (*T. gondii*, *Cytomegalovirus*, Epstein-Barr virus, *Brucella* species, HBV, HCV, HIV, *T. pallidum*, *Salmonella* spp). Full blood cell count was normal. Chest X-ray did not show any abnormalities. Lymph node US documented four lymph nodes at left axillary region with thickened wall, with diameters ranged from 1.8 to 3.5 cm. Also, many and small lymph nodes were found in submandibular, lateral cervical and groin regions. We decided to perform both serology and RT PCR for *Bartonella* spp. with the suspicion of CSD, finding positive IgG, IgM and RT PCR for *B. henselae*. So we administered IV ceftriaxone 1 g daily for a week and doxycyclin 100 mg twice daily *per os* for 6 weeks. After the first two weeks of treatment, symptoms disappeared and the patient referred well-being status. Also, in this case, RT PCR was useful for two reasons: firstly to evaluate if positivity was present, secondly to evaluate the efficacy of treatment in order to evaluate therapy interruption.

## 4 DISCUSSION

Clinical evolutions of diseases due to *Bartonella species* are diverse depending on bacterial species and status of the immune system of the host. In fact, infections can be mild or asymptomatic in immunocompetent hosts or severe in the immunocompromised hosts [5]. Moreover, no clinical trials or databases of clinical studies with standard case definitions, microbiological confirmation through culture, and rigidly defined disease outcomes are available. For these reasons, therapy indications are based only on case reports with a very limited number of subjects enrolled [1, 2]. In particular, treatment is not standardized, both for type of drugs and duration of therapy [2]. Indeed, few studies evaluated antibiotic schemes for CSD and chronic bacteriaemia [8]. For this reason, we tried to tailor therapy based on clinical pictures of individual patients.

For instance, case #2 was the most severe, so we added ceftriaxone to gentamicin and doxycycline for additive or synergistic effect. Such strategy appeared to be successful over a short-term follow-up as demonstrated by sustained undetectability of *Bartonella* DNA.

Since *Bartonella* are intracellular bacteria, their isolation through culture is very difficult and slow, even on cell systems [6]. So, bartonelloses are often diagnosed through serological tests. However, these tests can be negative since the conventional anti-*Bartonella henselae* IgM ELISA methods are poor for as far as both sensitivity and specificity are concerned [7]. In line with data showing that serology is affected by false negative results, among our patients, only 1 out of 3 had positive IgM, while Bartonella DNA was positive in all cases. On the other hand, it should be considered that the examined patients showed clinical signs started long time before, so it is not unusual to obtain negative IgM because this kind of antibodies may have already disappeared. By contrast, if IgM had disappeared, IgG were positive in all examined cases, a result concordant with the positivity of RT PCR. Moreover, not only RT PCR was useful for diagnostic purposes, but also helped significantly for treatment monitoring and decisions regarding therapy continuation or interruption. Indeed, even if drug regimens were heterogeneous because they were tailored according to the severity of the clinical picture, RT PCR was consistent with the clinical course of patients, indicating to stop therapy after *Bartonella* DNA was found to be persistently negative. Given the lack of precise standards for treatment, this potential of RT PCR merits to be emphasized to clinicians.

## CONCLUSION

 In conclusion, this case series indicated the utility of molecular identification and quantification of *Bartonellae* spp. infections with species-specific RT PCR both for diagnosis and monitoring of treatment response. Further studies are necessary to validate these results and elucidate what is the best treatment for this pleomorphic disease.

## Figures and Tables

**Fig. (1):**
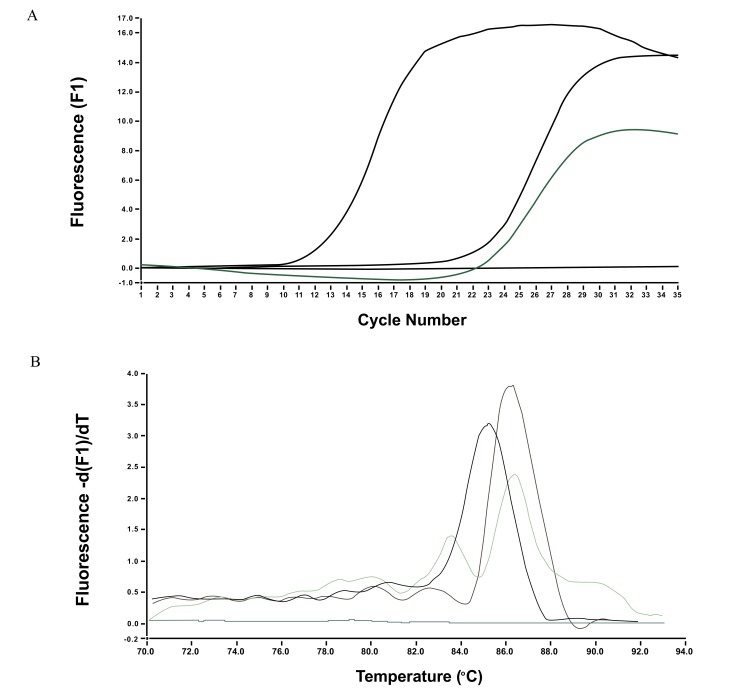
Amplification and melting curve of *B. henselae* (obtained from case report #2). Real time lyght cycler PCR assay of *Bartonella *spp. (A). Identification of PCR products was confirmed by melting curve analysis. The melting curve profiling revealed specific DNA of *B. henselae* in blood sample (B).

**Table 1:** Laboratory and clinical course of patient #1.

Date	IgG (titres)	IgM (titres)	RT PCR DNA	Therapy	Signs or Symptoms
July, 24^th^ 2013	Positive 1:320	Negative	Not done	No therapy	Asthenia, lymphadenopathy
October, 31^rst^ 2013	Positive 1:320	Negative	Positive	No therapy	Asthenia, lymphadenopathy
February, 11^th^ 2014	Positive 1:320	Negative	Positive	Doxycycline 100 mg twice daily, *per os,* for ten days	Asthenia, lymphadenopathy
March, 12^th^ 2014	Not done	Not done	Positive	Doxycycline 100 mg twice daily, *per os,* for two weeks	Asthenia, lymphadenopathy
April, 16^th^ 2014	Not done	Not done	Negative	Doxycycline 100 mg twice daily, *per os,* for two weeks	Lymphadenopathy
May, 13^th^ 2014	Not done	Not done	Negative	No therapy	Small center latero-cervical node
June, 9^th^ 2014	Not done	Not done	Negative	No therapy	Small center latero-cervical node
January, 27^th^ 2015	Not done	Not done	Negative	No therapy	No signs or symptoms

**Table 2:** Laboratory and clinical course of patient #2.

Date	IgG (titres)	IgM (titres)	RT PCR	Therapy	Signs or Symptoms
August, 7^th^ 2013	Positive 1:320	Negative	Not done	No therapy	Lymphadenopathy, recurrent fever, chilling and malaise
October, 29^th^ 2013	Positive 1:320	Negative	Positive	No therapy	Lymphadenopathy, recurrent fever, chilling and malaise
December, 10^th^ 2013	Not done	Not done	Positive	Gentamicin 80 mg three times *per* day IV, and Ceftriaxone 1 g twice daily IV, for a week and Doxycycline 100 mg twice daily, *per os,* for six weeks	Lymphadenopathy, recurrent fever, chilling and malaise
December, 20^th^ 2013	Not done	Not done	Negative	Doxycycline 100 mg twice daily, *per os,* for four weeks and four days	Lymphadenopathy
January, 29^th^ 2014	Positive 1:320	Negative	Negative	No therapy	No signs or symptoms
March, 4^th^ 2014	Not done	Not done	Negative	No therapy	No signs or symptoms

**Table 3:** Laboratory and clinical course of patient #3.

Date	IgG (titres)	IgM (titres)	RT PCR	Therapy	Signs or Symptoms
October, 17^th^ 2014	Positive 1:320	Positive	Positive	Ceftriaxone 1 g twice daily IV, for a week and Doxycycline 100 mg twice daily, *per os,* for six weeks	Lymphadenopathy, arthralgia, asthenia, and low-grade fever
December, 12^th^ 2014	Not done	Not done	Negative	No therapy	No signs or symptoms
